# Youth on Board: Redefining Health Research for True Adolescent Involvement

**DOI:** 10.1111/hex.70016

**Published:** 2024-09-30

**Authors:** Alina Yang

**Affiliations:** ^1^ Department of Public Health Scarsdale High School Scarsdale New York USA

Dear Editor,

I read with great interest the review article entitled “A Rapid Review of Guidelines on the Involvement of Adolescents in Health Research” [1]. The authors highlight the importance of meaningful adolescent participation in research, a principle I strongly endorse as a high school advocate engaged in cardiovascular disease (CVD) research. Adolescents, as a distinct and dynamic population, offer unique perspectives that are essential for the relevance and impact of health research, particularly in matters directly affecting them. However, as the review reveals, existing guidelines often fall short of providing comprehensive frameworks for youth involvement. This gap is concerning, given the potential for adolescents to significantly shape research outcomes and enhance the applicability of empirical findings.

Many of the current guidelines are developed with a narrow focus, overlooking the diverse needs and experiences of adolescents across different cultural, ethnic, socioeconomic and geographic contexts. Nevertheless, because research most often reflects the views of researchers themselves, we can streamline our workflow by increasing the involvement of youth from diverse contexts, thereby increasing the quality of research projects aimed at addressing the disparities in adolescent health across these contexts.

The call for a more systematic approach to guideline development is both timely and imperative: adolescents should not only be participants in research but also active contributors to the design of guidelines and studies that affect them. Only this way can we create guidelines and produce results that are truly representative of the adolescent experience. Moreover, through social media platforms and other recruitment strategies that resonate with youth, ethical considerations that protect their personal information, training programmes that equip them with the skills to contribute meaningfully to research and proper power dynamics, we can combat the current challenges faced by aspiring adolescent researchers [2].

Improved guidelines that facilitate purposeful adolescent participation in health research can not only tap into a fresher perspective in the field but also have a positive impact on the quality of research and methodological approaches [3]. Involving youth in research can help us understand their perceptions of public health and barriers to healthy behaviours, pointing us in the right direction of developing more effective prevention and intervention programmes while empowering them to become advocates for their own health and well‐being. This will simultaneously foster a new generation of young researchers, potentially accelerating advancements in the field of public health and ultimately leading to better outcomes for adolescents worldwide.

## Author Contributions

The author confirms sole responsibility for the preparation, writing and revision of this manuscript.

## Conflicts of Interest

The author declares no conflicts of interest.

## Data Availability

Data sharing is not applicable to this article as no new data were created or analysed in this study.
